# Preoperative transcutaneous vagus nerve stimulation as a novel strategy to prevent postoperative atrial fibrillation in calcific aortic valve disease: mechanistic insights and translational perspectives

**DOI:** 10.3389/fcvm.2025.1625436

**Published:** 2026-01-23

**Authors:** Justine Bergeon, Fanette Chassagne, Marie Fanget, Angèle N. Merlet, Stéphane Avril, Léonard Féasson, Frédéric Roche, Magnus Bäck, David Hupin

**Affiliations:** 1 Translational Cardiology, Department of Medicine Solna, Center for Molecular Medicine, Karolinska Institutet, Stockholm, Sweden; 2 Mines Saint-Etienne, University Jean Monnet, INSERM, U 1059, Sainbiose, Saint Etienne, France; 3 Univ Jean Monnet, Department of Clinical and Exercise Physiology, University Hospital of Saint-Etienne, Mines Saint-Étienne, INSERM U 1059, Saint-Étienne, France; 4 Univ Jean Monnet, Department of Clinical and Exercise Physiology, University Hospital of Saint-Etienne, LIBM, Saint-Étienne, France; 5 Université de Lorraine, Inserm, DCAC, Nancy, France

**Keywords:** autonomic nervous system, calcific aortic valve stenosis, inflammation, postoperative atrial fibrillation, transcutaneous vagus nerve stimulation

## Abstract

Postoperative atrial fibrillation (POAF) affects 38%–63% of patients undergoing surgical replacement for calcific aortic valve stenosis (CAVS), increasing morbidity, stroke risk, and hospital stay. POAF results from an interplay between pre-existing arrhythmogenic substrates, acute surgical triggers, unresolved inflammation, and autonomic nervous system (ANS) imbalance. Specialized pro-resolving mediators (SPMs) orchestrate inflammation resolution and tissue homeostasis; their deficiency may sustain valvular inflammation and promote arrhythmogenesis. Transcutaneous vagus nerve stimulation (tVNS) is a non-invasive approach that enhances parasympathetic tone, restores sympathovagal balance, and modulates inflammatory pathways. While tVNS has been applied postoperatively, its preoperative, preventive use in POAF has not been explored, representing a novel therapeutic strategy. In patients with CAVS, preoperative tVNS could reduce POAF by regulating ANS activity and limiting perioperative inflammation. Mechanistic insights may be gained through perioperative sampling, analysis of excised valvular and atrial tissue, and biomechanical assessments comparing stimulated and control groups. Preoperative tVNS thus offers a promising strategy to prevent POAF while addressing valvular inflammation, bridging translational physiology with clinical cardiology and potentially opening new avenues for the management of CAVS.

## 1 Introduction

Cardiovascular disease remains the leading cause of death worldwide (1). Despite remarkable advances in surgical and pharmacological therapies, the aging of the population continues to drive the burden of cardiovascular morbidity (2). Beyond atherosclerosis, conditions such as valvular heart disease and cardiac arrhythmias increasingly share a strong inflammatory component (3). Among them, calcific aortic valve stenosis (CAVS) stands out as a progressive disorder marked by chronic inflammation and active calcification processes that gradually impair valve function (3).

POAF remains the most frequent arrhythmic complication following cardiac surgery, affecting nearly half of patients undergoing aortic valve replacement. POAF significantly prolongs hospitalization and increases the risk of stroke and mortality (4). Although traditionally attributed to mechanical stress and atrial remodeling, growing evidence suggests that inflammation and autonomic nervous system (ANS) imbalance play key pathophysiological roles in its onset (5–7).

These converging findings suggest a shared inflammatory and autonomic substrate between valvular calcification and atrial arrhythmogenesis, opening a unique therapeutic window for neuromodulatory interventions. Transcutaneous vagus nerve stimulation (tVNS), a non-invasive, easily applicable technique, has recently shown promise in reducing inflammation and restoring autonomic balance (8, 9). However, while tVNS has been explored for preventing POAF, its potential impact on valvular inflammatory mechanisms remains unexplored.

This mini-review synthesizes recent insights linking inflammation, ANS dysfunction, and cardiac surgery–related arrhythmias, while discussing the therapeutic rationale for tVNS in preventing POAF and modulating inflammation in CAVS.

### 1.1 Atrial fibrillation and inflammation

Inflammation is now recognized as a key driver of both atrial and valvular disease progression. In the postoperative setting, inflammatory responses interact with autonomic imbalance to promote POAF (6). In patients undergoing valve replacement for severe calcific aortic stenosis, the incidence of POAF reaches 38%–63% (5) and up to 52% in the Swedish DAVAACA cohort (10). At the molecular level, inflammation and autonomic imbalance are tightly interwoven. The stress of surgery and transient periods of reduced perfusion lead to cytokine release and activation of systemic inflammatory cascades, including IL-6, TNF-α, and CRP (11). In parallel, perioperative stress enhances sympathetic activity and suppresses parasympathetic tone, further amplifying the inflammatory response and creating a substrate for postoperative arrhythmias (4, 5, 12). Conversely, parasympathetic activation through the vagus nerve promotes anti-inflammatory responses via the cholinergic anti-inflammatory pathway (13).

A central concept emerging in this field is the resolution of inflammation, an active process orchestrated by specialized pro-resolving mediators (SPMs) such as resolvins and protectins (14, 15). These lipid mediators limit leukocyte infiltration and promote tissue repair, restoring homeostasis after injury. In the postoperative heart, insufficient resolution responses may sustain low-grade inflammation, thereby perpetuating arrhythmogenic remodeling (4, 5, 14). Enhancing these resolution mechanisms, potentially through vagal stimulation, represents a novel anti-inflammatory and cardioprotective strategy (6, 14–16).

Clinical and experimental studies support this idea; tVNS administered for two weeks after cardiac surgery significantly reduced POAF incidence (17). Mechanistically, this effect was associated with decreased macrophage infiltration, elevated acetylcholine levels, and activation of *α*7-nicotinic acetylcholine receptors (*α*7AChR), leading to suppression of TNF-α and CRP. Other studies indicate that tVNS activates brainstem nuclei such as the spinal trigeminal nucleus (Sp5) and subfornical organ (SFO), modulating cardiac vagal tone and sympathetic output (18).

Beyond inflammation control, vagal stimulation influences atrial electrophysiology and structural remodeling. It preserves connexin proteins Cx40 and Cx43, which are essential for maintaining intercellular electrical coupling and preventing conduction heterogeneity (19–22). Through activation of the JAK2–STAT3 pathway and inhibition of NF-*κ*B, tVNS exerts downstream anti-inflammatory and antioxidant effects (23, 24).

Genetic and biomarker data reinforce the causal link between inflammation and POAF. IL-6 gene polymorphisms (e.g., 147 G/C) influence postoperative cytokine levels and susceptibility to arrhythmia (25, 26). Elevated IL-6 in the early postoperative period strongly correlates with POAF onset (4, 26). Similarly, high postoperative CRP levels predict recurrence and duration of AF episodes (4, 5, 11, 25).

In summary, POAF can be viewed as the clinical manifestation of a double imbalance: an excessive inflammatory response coupled with impaired autonomic regulation (4, 5, 27). Interventions such as tVNS, by modulating both inflammation and ANS tone, provide a mechanistically grounded opportunity to prevent this arrhythmia (6, 16–21, 24). Further research is needed to delineate the specific inflammatory mediators and patient subgroups most likely to benefit (20, 27, 28).

### 1.2 Transcutaneous vagus nerve stimulation

The concept of stimulating the vagus nerve to restore physiological balance is ancient, with auricular acupuncture used for over 3,000 years to modulate internal organ function through ear stimulation. Modern neuroanatomy has confirmed that the auricular branch of the vagus nerve, or Arnold's nerve, innervates specific regions of the external ear, particularly the tragus and cymba conchae (28, 29). Tekdemir's landmark study in 1998 provided the anatomical basis for tVNS, a non-invasive technique activating the vagus nerve through cutaneous electrodes placed on the ear (30).

Stimulation of the auricular branch of the vagus nerve triggers an auriculocardiac reflex, capable of transient bradycardia when the ear is stimulated (29, 31). This reflex illustrates the intimate connection between auricular sensory pathways and cardiac autonomic regulation. Interestingly, patients with angina or myocardial infarction sometimes report ear discomfort, further supporting a cardiac–auricular axis (31).

Functional neuroimaging studies show that tVNS activates the nucleus tractus solitarius (NTS), a critical brainstem relay for autonomic control. From the NTS, parasympathetic efferent signals project to cardiac centers, modulating heart rate variability and inflammatory reflexes (14, 19, 31). Through this pathway, tVNS engages the cholinergic anti-inflammatory pathway, reducing systemic cytokine release and promoting immune homeostasis (31–34).

Unlike implanted vagus nerve stimulators, tVNS is safe, non-invasive, with adverse effects generally mild and transient, including local skin irritation, tingling, erythema or discomfort at the stimulation site, and occasionally brief dizziness or headache (vasovagal responses are rare) and easily repeatable, making it particularly suitable in perioperative and geriatric settings (35). tVNS enhances parasympathetic tone and attenuates sympathetic overactivity, a key driver of postoperative complications (8, 9, 34).

Aging and surgery disturb this delicate autonomic balance. Reduced vagal responsiveness and sympathetic predominance favor autonomic rigidity (9, 34, 36), and surgical stress amplifies these effects (4, 5). Together, these factors create a permissive environment for POAF. Restoring this equilibrium via tVNS represents a physiologically coherent therapeutic goal (6, 19, 20, 27).

### 1.3 tVNS and postoperative inflammation

Preclinical and clinical data highlight the anti-inflammatory and anti-arrhythmic potential of tVNS. Low-intensity stimulation protocols (20–30 Hz, below pain threshold) improve sympathovagal balance, increase heart rate variability, and reduce systemic inflammation (8, 9, 22, 37). In anesthetized dogs, chronic low-level tVNS reduced AF inducibility, likely through improved vagal modulation and decreased atrial oxidative stress (36).

tVNS activates central and peripheral immune pathways:

- Central: brainstem nuclei (e.g., NTS, locus coeruleus) and hypothalamic centers regulating sympathetic output (16, 19, 31).
- Peripheral: inhibition of macrophage cytokine production (TNF-α, IL-1β) via *α*7AChR activation (13, 16, 23, 33).

This signaling promotes the resolution of inflammation, limits tissue damage, and fosters healing (7, 15, 16, 32, 33). Translationally, these effects may reduce systemic inflammation predisposing to POAF and modulate local inflammation in the resected calcified aortic valve, preserving both rhythmic and structural cardiac integrity (6, 24, 27).

### 1.4 Future clinical trials direction

POAF remains one of the most challenging complications after surgical replacement for CAVS (4, 5). Despite optimized perioperative care, preventive strategies rely mainly on pharmacological interventions and remain only partially effective. tVNS, a non-invasive and easily applicable technique, has emerged as a promising approach to enhance parasympathetic tone, restore autonomic balance, and attenuate systemic inflammation (2, 6, 8, 24).

As shown in Table 1, existing studies indicate that tVNS has been evaluated almost exclusively in peri- or postoperative settings (6, 17, 27, 37–42). Several randomized trials demonstrated reductions in POAF incidence or AF burden, accompanied by decreases in inflammatory markers (TNF-α, CRP, IL-6). Postoperative RCTs by Andreas et al. (17) and Stavrakis et al. (37, 42) showed significantly lower POAF rates, and the ongoing STOP_AF trial (*NCT04514757*) extends these findings to broader surgical cohorts. Systematic reviews (38–40) consistently highlight sympathovagal rebalancing, activation of the cholinergic anti-inflammatory pathway, and improved atrial electrophysiology as key mechanisms. Acute neuromodulation data (41) further confirm immediate effects on atrial conduction, reinforcing the biological plausibility of vagal modulation in the surgical setting.

**Table 1:** Summary of clinical trials and key published studies investigating transcutaneous or low-level vagus nerve stimulation (tVNS/LLVNS) in atrial fibrillation (AF) and postoperative atrial fibrillation (POAF).

Studies/clinical trials	Number of patients	Population/condition	Type of VNS (protocol)	Study design	Main outcomes/findings
NCT04514757, “STOP_AF”	77	Post-operative cardiac surgery including valve replacement or repair	taVNS, LLVNS parameters, 20 Hz, 250 ms, 2 × 1 h/day	RCT, double-blind, sham-controlled	Primary outcome: POAF incidence Secondaries: days of hospitalization, inflammatory markers, Sympathetic neural markers
Ballas et al., 2025 (38)	—	POAF after cardiac surgery	—	Review	Highlights the central role of inflammation, oxidative stress and ischemia-reperfusion, supports biomarker selection (CRP, IL-6)
Zafeiropoulos et al., 2024,2022 (27, 39)	—	POAF after cardiac surgery, cardiovascular disease and AF	—	Review	Consolidated RCTs (NCT03392649, NCT04514757), trend toward ↓ POAF and shorter stay Mechanisms: vagal-sympathetic rebalancing, anti-inflammator*y* axis, repolarization stability
Bazoukis et al., 2023 (40)	—	Cardiovascular/AF models	—	Review	tVNS activates cholinergic anti-inflammatory pathway (↓ cytokines, less atrial remodeling)
Kharbanda et al., 2023 (41)	10	LLVNS model	LLVNS (tragus), 20 Hz, acute=1 min, chronique >20 min	-	↑ unipolar potential voltage; ↓ total activation time; ↑ slope of unipolar potentials; ↓ fractionation; change in sinoatrial node exit sites
Stavrakis et al. 2020 (42)	53	Paroxysmal AF	LLVNS (tragus), 20 Hz, 1 h/day for 6 months	RCT, double-blind, sham-controlled	↓ 75% AF burden after combining across the 3- and 6-month time points in the active compared to control group (*p* = 0.016) ↓ 23% TNF-α level (*p* = 0.0093)
Andreas et al., 2019 (17)	40	Cardiac surgery patients (CABG ± valve; POAF prevention)	taVNS, 1 Hz, 1 mA, 40 min ON/20 min OFF, up to 2 weeks post-op	RCT, double-blind, sham-controlled	↓ POAF incidence (20% vs. 55%; *p* = 0.022)
Stavrakis et al. 2017 (6)	54	POAF	LLVNS (vagus nerve preganglionic fibers alongside the lateral aspect of the superior vena cava), 20 Hz, 0.1 ms, 72 h post operation	RCT, sham-controlled	↓ POAF burden (12% vs. 36%, *p* = 0.027) ↓ inflammatory cytokines during postoperative hospitalization
Stavrakis et al. 2015 (37)	40	Paroxysmal AF	LLVNS (tragus), 20 Hz, 1 ms square wave, 1h	RCT, sham-controlled	↓ pacing-induced AF burden ↓ TNF-α (*p* = 0.006) and CRP (*p* = 0.001)

The table highlights study designs, patient populations, stimulation protocols, and main findings from randomized controlled trials and review articles.

AF, atrial fibrillation; POAF, postoperative atrial fibrillation; VNS, vagus nerve stimulation; tVNS, transcutaneous vagus nerve stimulation; taVNS, transcutaneous auricular vagus nerve stimulation; LLVNS, low-level vagus nerve stimulation; RCT, randomized controlled trial; CABG, coronary artery bypass graft; HFpEF, heart failure with preserved ejection fraction; TNF-α, tumor necrosis factor alpha; CRP, C-reactive protein; IL-6, interleukin-6; IL-8, interleukin-8.

**Figure 1:**
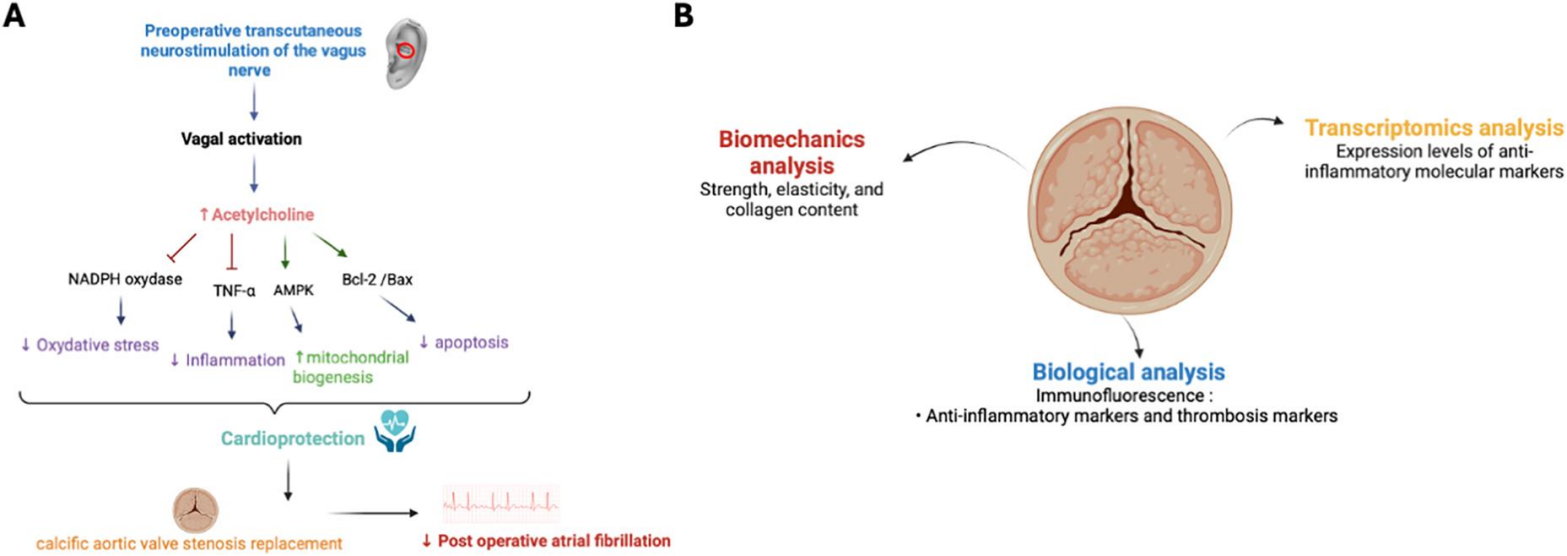
Mechanisms and translational analysis of cardioprotection and calcific aortic valve stenosis (CAVS). **(A)** Mechanisms of cardioprotection induced by preoperative transcutaneous vagus nerve stimulation (tVNS) in CAVS replacement. Increasing vagal tone through vagus nerve stimulation (tVNS), physical exercise, and/or pharmacological agents offers various cardioprotective benefits. These include antioxidant, anti-inflammatory, and anti-apoptotic effects, alongside the regulation of mitochondrial biogenesis and mitophagy. Arrows denote activation, while T-bars signify inhibition. *AMPK stands for AMP-activated protein kinase, TNF-*α *refers to tumor necrosis factor-*α *and nicotinamide adenine dinucleotide phosphate*. **(B)** Translational analysis of calcific aortic valve stenosis (CAVS). Illustration of the multimodal approach used to investigate calcific aortic valve stenosis (CAVS). Biomechanical analysis assesses strength, elasticity, and collagen content of the calcified valve. Biological analysis, including immunofluorescence, focuses on detecting anti-inflammatory and thrombosis markers. Transcriptomic analysis evaluates the expression levels of molecular markers associated with inflammation and thrombosis.

Importantly, no study to date has evaluated preoperative tVNS, underscoring the novelty of the preventive approach discussed here. Preoperative neuromodulation could stabilize autonomic tone, reduce the perioperative cytokine surge, and enhance myocardial resilience before surgery begins (13, 16, 21). In patients with CAVS who exhibit chronic inflammation and ANS dysfunction, this strategy may have dual benefits by reducing POAF incidence while modulating valvular inflammatory activity (Figure 1A). In addition to tVNS, other autonomic neuromodulatory interventions, such as stellate ganglion blockade, modulation of ganglionated plexi, or renal denervation have been explored to reduce POAF (39, 43). While some demonstrated reductions in atrial vulnerability, these techniques are invasive or technically demanding, limiting their perioperative use. In this context, tVNS uniquely combines non-invasiveness, safety, and the ability to target the same autonomic and inflammatory pathways, strengthening its potential as a practical preventive strategy.

Key questions for future clinical trials include:

− Identification of patient subgroups (e.g., those with elevated inflammatory or autonomic risk profiles) most likely to benefit from preoperative tVNS;
− Determination of the optimal stimulation parameters (frequency, intensity, duration) and timing relative to surgery;
− Evaluation of perioperative biomarkers such as CRP, IL-6, or SPMs as indicators of therapeutic response (7, 14, 15, 25, 26)?

Mechanistic exploration should integrate clinical outcomes with perioperative biomarker profiling, and analysis of excised aortic valves and atrial tissues, complemented by biomechanical and molecular assessments to delineate tVNS-mediated cardioprotective and anti-inflammatory effects (Figure 1B) (3, 14, 15). Such a multimodal, translational strategy could illuminate how autonomic neuromodulation regulates inflammation resolution, tissue remodeling, and valvular homeostasis.

In summary, preoperative tVNS emerges as a physiologically coherent and clinically testable strategy to prevent POAF and modulate valvular inflammation in CAVS. By bridging fundamental mechanisms with clinical outcomes, forthcoming trials could establish non-invasive autonomic neuromodulation as a new paradigm in perioperative cardiovascular protection and disease modification (2, 6, 17, 24, 27).
